# Contrast-associated acute kidney injury: does it really exist, and if so, what to do about it?

**DOI:** 10.12688/f1000research.16347.1

**Published:** 2019-05-29

**Authors:** Wim Vandenberghe, Eric Hoste

**Affiliations:** 1 Department of Intensive Care Medicine, University Hospital Ghent, Ghent University, C. Heymanslaan 10, 9000 Ghent, Belgium; 2 Research Foundation-Flanders (FWO), Egmontstraat 5, 1000 Brussels, Belgium

**Keywords:** Acute kidney injury, Contrast media, Coronary angiography, Angiography, Tomography X-Ray Computed, Intensive Care Unit

## Abstract

For decades, when contrast agents are administrated, physicians have been concerned because of the risk of inducing acute kidney injury (AKI). Recent literature questions the existence of AKI induced by contrast, but animal studies clearly showed harmful effects. The occurrence of contrast-associated AKI was likely overestimated in the past because of confounders for AKI. Several strategies have been investigated to reduce contrast-associated AKI but even for the most important one, hydration, there are conflicting data. Even if the occurrence rate of contrast-associated AKI is low, AKI is related to worse outcomes. Therefore, besides limiting contrast agent usage, general AKI preventive measurements should be applied in at-risk patients.

## Introduction

Iodinated radiocontrast agents are used for diagnostic radiography procedures as well as for therapeutic interventions. With the increasing use of non-invasive endovascular interventions, use of and exposure to contrast agents will further increase. Over decades, physicians were concerned about the harmful effects of contrast agents on kidney function. Recently, this fear led to doubts about whether contrast-associated acute kidney injury (CA-AKI) even exists and, if so, what is the clinical impact and are we able to prevent it?

## Does contrast-associated acute kidney injury even exist?

Maybe the best illustration of the different opinions on the impact of contrast exposure on AKI is the use of many different terms. Classically, the term contrast-induced AKI was used; later, others suggested the terms CA-AKI and post-contrast AKI ^1, 2^. Contrast-induced AKI indicates a clear relationship between contrast administration and AKI. The latter two terms better illustrate the heterogeneity and multifactorial etiology of AKI in severely ill patients exposed to contrast agents. Post-contrast AKI emphasizes the temporal relationship between contrast exposure and AKI.

**Figure 1.**
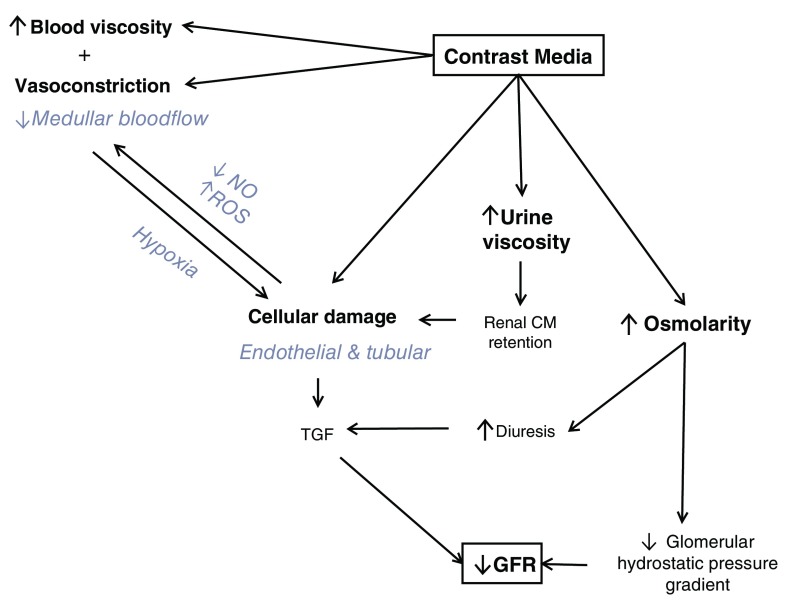
Pathophysiology of contrast-associated acute kidney injury. CM, contrast media; GFR, glomerular filtration rate; NO, nitric oxide; ROS, reactive oxygen species; TGF, transforming growth factor. Modified version from Vandenberghe W, De Corte W, Hoste EA. Contrast-associated AKI in the critically ill: relevant or irrelevant? Current opinion in critical care. 2014 Dec;20(6):596-605. PubMed PMID: 25314241. Epub 2014/10/15 ^9^. By permission of Wolters Kluwer Health, Inc.

In experimental studies, it is clearly proven that contrast agents reduce renal blood flow in the medulla, induce free oxygen radicals, and induce apoptosis of renal tubular cells ( Figure 1) ^3– 5^. Several trials have evaluated various strategies to prevent AKI after exposure to contrast agents. Even for the most accepted and most often used preventive measure, pre-hydration, there are conflicting data. Negative trials for preventive measures and conflicting data in controlled cohort studies may be an argument against a causative relationship between contrast agents and AKI ^6^. Bias may occur in studies if the investigated preventive measure influences creatinine concentration even without effect on the contrast agent or its potential effects on kidney function. For example, *N*-acetylcysteine is associated with lower creatinine production, dialysis removes creatinine, and hydration can lead to dilution of creatinine ^7^. On the other hand, in human studies, high-osmolar contrast agents were found to be harmful for kidneys and therefore are not used anymore ^8^. The ultimate evidence on the toxicity of contrast administration would be a prospective randomized study in which patients who underwent, for example, a computed tomography (CT) scan would be randomly assigned to a contrast-enhanced examination or standard. **So far, such a study does not exist**. Sophisticated statistical analyses may be used in cohort studies to account for differences in baseline characteristics and other confounders but these studies will always suffer from bias by unmeasured confounding.

Given that experimental data clearly demonstrate the toxic effects of contrast exposure on kidney function, we prefer to use the terminology CA-AKI throughout this article.

In severely ill patients with AKI, it is difficult to differentiate the role of contrast agents and other possible contributors to the development of AKI, such as hypotension, infection, inflammation, and other nephrotoxic medications ^10^. To complicate things even more, even the procedure itself can have an influence on kidney function. During percutaneous coronary intervention (PCI), passage of the guidewire and catheter may disrupt plaques in the aorta, leading to micro-embolism and cholesterol emboli in the kidneys via the renal arteries ^11^.

Another important issue is the definition used to diagnose CA-AKI. Chalikias *et al*. ^12^ found that six different definitions were used in recent literature. Some used an absolute increase of serum creatinine which varied from at least 0.3 to at least 1 mg/dL, and others used relative increases of 25 to 50% of baseline creatinine. Also, the duration of observation was different: classically a 48-hour follow-up time after contrast exposure and up to 6 days in the AMACING trial ^12, 13^. Chalikias *et al*. showed a wide variation of the occurrence rate of CA-AKI, between 1.3 and 15.8%, probably because of the use of different definitions together with differences in the patient cohort undergoing angiography ^12^. We investigated the occurrence rate of CA-AKI in intensive care unit (ICU) patients after contrast administration for CT scan and non-coronary angiography and found that the occurrence rate of CA-AKI varied between 16.3 and 22.2% when CA-AKI was defined respectively as an increase of serum creatinine of 25 or 0.5 mg/dL within 3 days or the Kidney Disease Improving Global Outcomes (KDIGO) classification for AKI ^1^.

## Is it possible to prevent contrast-associated acute kidney injury?

We should realize that there is important heterogeneity between patient groups exposed to contrast. Some groups are at greater risks than others and this also may explain the discussion on the relevance of CA-AKI. The risk for CA-AKI will be different in out-patients receiving contrast for coronary angiography compared with ICU patients undergoing a contrast-enhanced CT scan. In patients who undergo coronary angiography, exposure to contrast is in most cases the single reason for AKI, whereas in critically ill patients who are having a contrast-enhanced CT scan, AKI is more likely the result of multiple hits. In fact, a contrast-enhanced CT scan is typically carried out in patients who are at greater risk for AKI. An illustrative example may be a patient with a suture leak one week after colectomy for colon cancer who develops septic shock. In this case, there are many risk factors besides contrast: previous major abdominal surgery, septic shock, and use of broad-spectrum antibiotics, including aminoglycosides and vancomycin. Here, simple preventive measures may make an important impact ^14, 15^.

## Who should receive contrast prevention?

Especially in out-patients with normal kidney function undergoing elective procedures, the risk for CA-AKI and long-term consequences is low. Preventive measures should be administered to patients at greatest risk for CA-AKI. Risk factors include decreased kidney function, as measured by an estimated glomerular filtration rate (eGFR) lower than 45 mL/min per 1.73 m ^2^, or the presence of other risk factors for AKI ( Table 1).

**Table 1.** Risk factors for contrast-associated acute kidney injury ^2^.

Risk factors for CA-AKI
Patient-related - eGFR < 30 mL/min per 1.73 m ^2^ before IV or IA CM administration (level C) - eGFR < 45 mL/min per 1.73 m ^2^ if patient admitted on ICU or if IA CM administration (level C) - Risk factors for impaired renal function in general (not specific for CA-AKI) (level B) ○ Old age ○ Female gender ○ Low body mass index ○ Cardiovascular and metabolic risk factors ○ Malignancy ○ Inflammation ○ Bleeding ○ Anemia ○ Hyperuricemia Procedure-related - Repeated CM injections in a short period (48–72 hours) (level C) - High CM dose (level C) ○ Ratio of CM dose to absolute eGFR should be < 1.1 ○ Ratio of CM volume to eGFR should be < 3.0 (if CM concentration is 350 mg iodine/mL)

CA-AKI, contrast-associated acute kidney injury; CM, contrast media; eGFR, estimated glomerular filtration rate; IA, intra-arterial; ICU, intensive care unit; IV, intravenous.

## Measures for contrast prevention

Several specific interventions to prevent CA-AKI have been investigated. We will discuss the use of hydration, bicarbonate, *N*-acetylcysteine, vitamins, statins, and dialysis.

### Hydration

Adequate hydration before and after contrast agent administration is regarded as the most important preventive strategy. Its rationale is that hydration leads to a lower concentration of contrast agent at the site of the renal tubules, which will reduce interaction of the contrast with the kidneys. Despite the wide adoption of hydration as a preventive measure for CA-AKI, this gets only level B evidence in the 2011 and 2018 guidelines for contrast medium–induced nephropathy by the contrast media safety committee of the European Society of Urogenital Radiology ^2, 16, 17^. Recently, the AMACING trial, one of the few studies that actually compared hydration with no hydration, could not show a benefit for hydration as a preventive measure for CA-AKI ^13^. In this study, patients who had an elective contrast-enhanced procedure and who had an eGFR lower than 60 mL/min per 1.75 m ^2^ but greater than 30 mL/min per 1.75 m ^2^, were prospectively included and allocated to a group with or without hydration ^13^. The AMACING trial included patients with reduced kidney function for whom hydration as a preventive strategy is recommended. Interestingly, the incidence of CA-AKI in this study cohort was less than 3% in both the intervention and control groups. Severely ill ICU patients who undeniably have a higher risk for the development of CA-AKI were excluded and therefore the results cannot be extrapolated to this population.

Hydration is also not without side effects and especially patients with reduced cardiac and kidney function are at risk of pulmonary edema. In this population, the volume of hydration is often reduced to prevent fluid overload, which of course may increase the risk of inadequate renal protection.

***Hydration with bicarbonate or isotonic saline?*** Hydration with isotonic saline has been the standard for over a decade in coronary angiography ^18^. Merten used bicarbonate as a scavenger for free reactive oxygen species in a solution with a concentration similar to that of NaCl 0.9% in order to protect kidneys against contrast ^19^. Follow-up studies in which a bicarbonate regimen was compared with isotonic saline showed conflicting results; some studies and meta-analyses confirmed the benefits shown by the original study by Merten and others showed equal risk for CA-AKI. Unfortunately, most of these studies had important limitations in study design/conduct and most importantly were underpowered, limiting their interpretation ^2, 20– 23^.

Recently, the adequately powered PRESERVE study, a prospective randomized study that had a 2 × 2 factorial design and that included 4993 patients with a high risk for renal complications after contrast administration, showed no benefit of intravenous sodium bicarbonate over intravenous sodium chloride for the prevention of death, need for dialysis, or persistent decline in kidney function at 90 days or for the prevention of CA-AKI ^24^. The absence of benefit for sodium bicarbonate compared with isotonic saline was confirmed in a prospective multicenter randomized study in an ICU setting in patients with stable renal function ^25^. Therefore, we should conclude that sodium bicarbonate solutions and isotonic saline are equally effective for prevention of CA-AKI in this type of patient.

### Diuretics

Some preventive strategies such as the use of the osmotic diuretic mannitol or the loop diuretic furosemide are aimed at a reduction of exposure of the tubular cells to contrast by increasing tubular flow ^26^. When increased urine production and negative fluid balance were not corrected, these strategies resulted in net fluid loss and so provided a nice model to show that volume depletion comes with increased risk for CA-AKI ^26^. The RenalGuard System uses forced diuresis by means of furosemide in combination with a device that provides continuous intravenous fluid compensation of the urine produced ^27^. This system prevented CA-AKI and fluid overload in three studies in patients who underwent coronary angiography or PCI with reduced cardiac function ^28, 29^.

### *N*-acetylcysteine

Similar to sodium bicarbonate pre-hydration, *N*-acetylcysteine has been explored for prevention of CA-AKI for the supposed anti-oxidant effects on reactive oxygen species ^30^. Since the original study by Tepel *et al*. ^30^ in 2000, numerous studies and meta-analyses, most underpowered and with methodological flaws, have reported conflicting results on the use of *N*-acetylcysteine for the prevention of CA-AKI. Recently, however, two large and adequately powered studies could show no benefit of *N*-acetylcysteine over placebo in different study cohorts. First, the ACT (Acetylcysteine for Contrast-Induced Nephropathy Trial), including 2308 patients in coronary angiography and peripheral vascular angiography, showed that at-risk patients exposed to high-dose *N*-acetylcysteine and placebo had similar rates of CA-AKI ^31^. Also, the PRESERVE study (n = 4998) showed that the two groups had similar incidences of CA-AKI ( *N*-acetylcysteine 9.1% versus placebo 8.7%, *P* = 0.58) ^24^. In summary, the supposed preventive effect of *N*-acetylcysteine for CA-AKI could not be confirmed in several well-designed and adequately powered studies.

### Statins

Statins have anti-oxidant, anti-inflammatory, and anti-thrombotic properties and restore renal nitric oxide (NO) production ^32^. These mechanisms play a role in CA-AKI and therefore several trials have investigated various types and doses of statins as a preventive measure ^33^. A meta-analysis containing 150 trials with several preventive strategies for CA-AKI found only a beneficial effect of statins on the general population ^34^. In 2017, Liang *et al*. performed a meta-analysis of 15 randomized controlled trials (RCTs) about moderate- to high-dose rosuvastatin for the prevention of CA-AKI after angiography or PCI ^35^. A moderate to high dose of rosuvastatin, compared with low-dose or no statin, reduced CA-AKI in that specific cohort ^35^. In the same year, an RCT with atorvastatin confirmed the benefit for reducing CI-AKI ^36^. Therefore, in patients undergoing coronary angiography, an expert panel of the European Society of Intensive Care Medicine suggests the short-term use of atorvastatin or rosuvastatin to prevent CA-AKI ^37^.

### Renal replacement therapy

Hemodialysis is able to remove between 70 and 80% of the injected low-osmolar contrast media (LOCM) dose during a 4-hour session ^38– 40^. Although it is technically possible to remove a certain amount of contrast dose, a 2012 meta-analysis by Cruz *et al*. showed insufficient evidence to support renal replacement therapy (RRT) to prevent CA-AKI ^41^.

A potential explanation for the discrepancy between the ability to remove contrast by RRT and the lack of effect in preventing CA-AKI is that RRT induces inflammation, coagulation, and hypotension that in themselves may negatively affect kidney function ^41^. Second, for practical reasons, there will inevitably already be a considerable time of contrast exposure between time of administration and start of RRT. Probably, the nephrotoxic effects of contrast are most important immediately after administration when contrast concentration in the renal arteries and kidneys is highest ^41^. Marenzi *et al*. randomized patients with severely impaired kidney function beforehand to initiation of hemofiltration before coronarography and continued afterwards and showed a less frequent rise in serum creatinine (which is not unexpected when RRT is used) and fewer complications in the intervention group compared to standard of care ^42^. Limitations of this study, including unblinding and a higher level of care in the intervention arm compared with control patients, mean that this intervention cannot be recommended at present.

### Vitamins C and E

Vitamins C and E have been evaluated as a preventive measure for CA-AKI for their anti-oxidant and scavenging effects. For vitamin C, encouraging results from cohort studies could not be confirmed in prospective studies ^43– 45^. Vitamin E was evaluated in four small prospective studies, and the individual studies and a meta-analysis (n = 623) showed that adding vitamin E to hydration regimens lowered the risk for CA-AKI ^46^. These encouraging results should be confirmed in adequately powered studies before wide adoption of this preventive strategy can be recommended.

## How can the radiologist or cardiologist make nice images without destruction of the kidneys?

### Route of administration

The risk for CA-AKI seems lower when contrast media is administered intravenously rather than intra-arterially ^16^. There are several explanations for this. During an intra-arterial contrast procedure, a higher dose is usually used and the transit time to the kidneys is shorter. The combination of these two factors will result in potentially higher concentrations of contrast medium in the kidneys. In addition, there are more procedure-related reasons for AKI. Advancing the catheter via the femoral artery to the aorta increases the risk for thrombo-emboly entering the renal arteries. The risk for CA-AKI is lower in coronary angiography via the radial artery compared with the femoral approach ^47^. This line of reasoning is merely theoretical since in clinical practice the route of administration—intravenous or intra-arterial—is procedure-related. Importantly, a higher risk for CA-AKI in patients with intra-arterial contrast may be explained by differences in baseline characteristics when compared with patients who undergo intravenous contrast examination. Both Kooiman *et al*. ^48, 49^ and McDonald *et al*. ^48, 49^ addressed this potential bias by investigating paired cohorts of patients receiving both intra-arterial and intravenous contrast. The authors were not able to show a difference in occurrence rate of CA-AKI between intra-arterial versus intravenous contrast administration ^48, 49^.

### Type of contrast medium

Contrast agents are categorized according to their osmolality as high-, iso-, or low-osmolar contrast media (HOCM, IOCM, and LOCM, respectively). Especially HOCM is a risk factor for CA-AKI and is not used anymore. Whether IOCM and LOCM agents have an advantage over each other is unclear. The Visipaque Angiography/Interventions with Laboratory Outcomes in Renal Insufficiency (VALOR) and Cardiac Angiography in Renally Impaired Patients (CARE) trials were not able to show a different in occurrence of CA-AKI when IOCM and LOCM were used in patients with chronic kidney disease undergoing angiography ^50, 51^. This was supported by a meta-analysis by Heinrich *et al*. ^52^. Similar results were found recently in a meta-analysis including prospectively randomized trials in diabetes patients, a high-risk group for CA-AKI ^53^.

### Volume of contrast agent

For every drug, too much is not good and will cause toxicity. Similarly, increased volumes of administered contrast will lead to a higher occurrence rate of AKI. Laskey *et al*. related volume of contrast to kidney function and found that a contrast volume equal to 3.7 times creatinine clearance was associated with an early abnormal increase in serum creatinine in patients undergoing PCI ^54^. This finding also nicely illustrates that patients may have different risk profiles. Patients with normal kidney function have a very low risk for CA-AKI whereas patients with decreased kidney function are at greater risk. A safe dose does not exist, and it is prudent to always use the smallest amount of contrast agent necessary to perform the investigation ^16^.

### Use of other imaging techniques: magnetic resonance imaging and gadolinium and carbon dioxide

Magnetic resonance imaging (MRI) could be an alternative for imaging in certain situations. Of course, a major disadvantage of MRI is the limited availability of this procedure. Also, early experience showed that gadolinium-based contrast agents (GBCAs) are associated with a decline in kidney function and evolution to nephrogenic systemic fibrosis (NSF). In 2017, Scharnweber *et al*. investigated the use of GBCA in patients with AKI, severe chronic kidney insufficiency, or even kidney failure with or without dialysis ^55^. Currently, according to their findings, macrocyclic agents and the newer linear agents (“cyclic” and “linear” are both chemical structures of GBCA) have a low risk of causing NSF when administered in a routine dose and when repeated injections are avoided ^55^.

In clinical practice, it is probably very seldom that a physician chooses MRI above CT solely for kidney-protective reasons and this is because of limited availability and the potential negative effects of MRI GBCAs on kidney function, especially in patients with chronic kidney disease. Carbon dioxide (CO _2_) is inexpensive and can be used in certain procedures as an alternative to iodinated contrast agents when MRI and gadolinium are used ^56^.

## What if, after all preventive measurements, contrast-associated acute kidney injury still occurs?

We found that in ICU patients CA-AKI was associated with longer length of stay, worse survival in hospital, and a higher mortality up to 1 year after contrast administration ^1^. When we reviewed literature for outcome after CA-AKI in different patient cohorts, we found conflicting results for mortality. We found that there was a greater risk for increased mortality in observational studies compared with a similar risk with or without contrast agent in matched case control studies ^9^. Difference in baseline characteristics probably plays an important role here. In clinical practice, we are less likely to order a contrast-enhanced CT scan in patients at greater risk for CA-AKI. Therefore, the patients we deny contrast, because of a high risk for AKI, will be categorized in the control group.

If AKI occurs after the administration of contrast, the same recommendations formulated by the KDIGO in preventing AKI can be used: evaluate whether it is possible to stop nephrotoxic agents such as aminoglycosides, vancomycin, non-steroidal anti-inflammatory drugs, angiotensin-converting enzyme inhibitor, and angiotensin receptor blocker; optimize hemodynamics and volume status; closely monitor kidney function and fluid balance; avoid hyperglycemia; avoid further exposure to contrast agents; and avoid gelatins, colloids, and chloride-rich solutions ^14, 15^.

## Conclusions

Contrast media will harm the kidneys through several pathophysiological mechanisms. However, the true impact of contrast administration on the occurrence of AKI is a matter of debate. AKI after contrast administration has a very low occurrence rate in low-risk patients and is frequent in critically ill at-risk patients. In this last group, other mechanisms may also play a role in the development of AKI. Therefore, the term CA-AKI is preferred. CA-AKI is associated with worse outcomes. Avoiding or minimizing the volume of contrast media is recommended, and general preventive measurements for AKI should be applied in patients with risk factors. Pre-hydration is the preferred preventive measure that should be used in patients with risk factors for the development of CA-AKI. In coronary angiography, a high dose of statins has been shown to diminish the risk for CA-AKI.
